# The Prevention of Colliery Explosions

**Published:** 1896-08-01

**Authors:** 


### The Prevention of Colliery Explosions.

The recent discussion in Parliament on the Coal Mines Regulation Bill has again drawn attention to the oft-disputed point as to the best means of preventing those disastrous explosions which from time to time draw from all classes expressions of sympathy and commiseration for the lot of the coal miner.

As in so many other circumstances, we have here to strike a balance, and while doing what is possible to prevent these occasional great disasters, which, almost alone, draw the attention of the general public to the coal miner and his ways, to avoid producing other evils which might in the aggregate lead to far greater loss of life, and to far greater suffering and distress, than occurs from the aggregate of colliery explosions.

As is now well known, coal dust has a great deal to do with the production of the more destructive explosions in coal mines. Before this was recognised it was very difficult to understand the enormous and wide-spread destruction sometimes produced by these catastrophes. But when it was shown that a mere mechanical mixture of coal dust and air would explode the matter was explained, although the prevention of the explosion was by no means simplified.

More evil than the mere extension of the area involved is, however, produced by coal dust. When the air is charged with this dust it often contains an excess of carbon, and this not being fully oxidised on explosion, the product is carbonic oxide, a deadly poison. In every way, then, coal dust is a cause of disaster. It promotes explosion, it widens its effects, and it poisons those within its area. Yet the prevention of it is an extremely difficult matter. By every stroke of the miner's pick as he "holes" the coal, by every fall of coal, by every action, in fact, by which coal is touched with tools, dust is produced; so that ultimately every ledge and cranny in the mine becomes a lodgment for an accumulation which only requires to be dry and to be shaken to create an explosive mixture. The obvious suggestion is to water the mine, and, in fact, up to the present time sweeping and watering are the only known means of lessening the danger from the presence of coal dust in mines.

It is important, however, to bear in mind that the production of such a dampness of atmosphere as to prevent the drying of the dust is itself likely to be productive of diseases which, if not so sensational in their *dénouement*, at the least tend to be quite as fatal in the end. It is perfectly plain that no mere watering of visible dust is likely to be of much service. The only way of preventing the dust from becoming dry is so to saturate the air with moisture as to prevent it from absorbing moisture, all of which means that the miner must work in an atmosphere approaching the saturation point. This brings us face to face with the real question, because there is plenty of experience as to the evil effects of working in a saturated atmosphere.

This is not the place for a history of the long struggles which led to the passing of the Cotton Cloth Acts, but it is to be noted that the conditions which led to their being passed bore a very striking analogy to those which may be produced by the watering of the mines. The exigencies of trade had made it necessary to introduce steam into the weaving sheds, and the result was the development of such an amount of chest disease, and especially of consumption, as to lead to the agitation which eventuated in the passing of the Cotton Cloth Acts, by which the amount of moisture in the weaving sheds was limited; and it is to be feared that the conditions produced by watering the mines would be very similar to those which were common in the weaving sheds of Lancashire before the legislature interfered for the protection of the workpeople.

To do real good the process must not only wet the dust, but must produce so moist an atmosphere so to prevent the drying of dust in inaccessible regions, and this may produce evils so far reaching in their effects upon health as to overbalance the lessened risk of death from colliery explosion. On the other hand, a severe explosion —even of gas alone at first—may dislodge sufficient coal to produce fresh coal dust even when all the older accumulation are quite damp, so that at the best watering would be but an uncertain remedy. There is a wide field yet open for the exercise of ingenuity in the devising of means for the prevention of explosions in coal mines.

